# Monitoring of minimal residual disease (MRD) is useful to predict prognosis of adult patients with Ph-negative ALL: results of a prospective study (ALL MRD2002 Study)

**DOI:** 10.1186/1756-8722-6-14

**Published:** 2013-02-06

**Authors:** Koji Nagafuji, Toshihiro Miyamoto, Tetsuya Eto, Tomohiko Kamimura, Shuichi Taniguchi, Takashi Okamura, Eiichi Ohtsuka, Takashi Yoshida, Masakazu Higuchi, Goichi Yoshimoto, Tomoaki Fujisaki, Yasunobu Abe, Yasushi Takamatsu, Shouhei Yokota, Koichi Akashi, Mine Harada

**Affiliations:** 1 Division of Hematology and Oncology, Department of Medicine, Kurume University School of Medicine, 67 Asahi-machi, Kurume 830-0011, Japan; 2 Department of Medicine and Biosystemic Science, Kyushu University Graduate School of Medical Sciences, Fukuoka, Japan; 3 Department of Hematology, Hamanomachi Hospital, Fukuoka, Japan; 4 Department of Hematology, HaraSanshin General Hospital, Fukuoka, Japan; 5 Department of Hematology, Toranomon Hospital, Tokyo, Japan; 6 Department of Hematology, Oita Prefectural Hospital, Oita, Japan; 7 Department of Hematology, Toyama Prefectural Central Hospital, Toyama, Japan; 8 Department of Hematology, Kyushu Kosei-Nenkin Hospital, Kitakyushu, Japan; 9 Department of Hematology, Kyushu Medical Center, Fukuoka, Japan; 10 Department of Hematology, Matsuyama Red Cross Hospital, Ehime, Japan; 11 Department of Medicine and Bioregulatory Science, Kyushu University Graduate School of Medical Sciences, Fukuoka, Japan; 12 Department of Oncology Hematology, Fukuoka University Hospital, Fukuoka, Japan; 13 Department of Hematology, Kyoto Prefectural University of Medicine, Kyoto, Japan; 14 Medical Center for Karatsu Higashimatuura Medical Association, Karatsu, Japan

**Keywords:** Acute lymphoblastic leukemia, Minimal residual disease, Hematopoietic stem cell transplantation, Adult

## Abstract

**Background:**

Allogeneic hematopoietic stem cell transplantation (HSCT) for patients with Philadelphia chromosome (Ph)-negative acute lymphoblastic leukemia (ALL) in first complete remission (CR1) is much more intensive than multi-agent combined chemotherapy, although allogeneic HSCT is associated with increased morbidity and mortality when compared with such chemotherapy. Minimal residual disease (MRD) status has been proven to be a strong prognostic factor for adult patients with Ph-negative ALL.

**Methods:**

We investigated whether MRD status in adult patients with ALL is useful to decide clinical indications for allogeneic HSCT. We prospectively monitored MRD after induction and consolidation therapy in adult patients with Ph-negative ALL.

**Results:**

Of 110 adult ALL patients enrolled between July 2002 and August 2008, 101 were eligible, including 59 Ph-negative patients. MRD status was assessed in 43 patients by the detection of major rearrangements in *TCR* and *Ig* and the presence of chimeric mRNA. Thirty-nine patients achieved CR1, and their probabilities of 3-year overall survival and disease-free survival (DFS) were 74% and 56%, respectively. Patients who were MRD-negative after induction therapy (n = 26) had a significantly better 3-year DFS compared with those who were MRD-positive (n = 13; 69% vs. 31%, p = 0.004). All of 3 patients who were MRD-positive following consolidation chemotherapy and did not undergo allogeneic HSCT, relapsed and died within 3 years after CR.

**Conclusions:**

These results indicate that MRD monitoring is useful for determining the clinical indications for allogeneic HSCT in the treatment of ALL in CR1.

## Background

Although more than 80% of adult patients with Philadelphia chromosome (Ph)-negative acute lymphoblastic leukemia (ALL) achieve complete remission (CR) with conventional induction therapy, their 5-year survival is only 30%–40%. Leukemia relapse is the most common cause of treatment failure in ALL [1-6]. Therefore, post-remission therapy is necessary and should be optimized in the treatment of adult ALL patients. If prognosis of patients with ALL in CR1 is estimated to be favorable, chemotherapy is usually continued to prevent leukemia relapse. However, patients with less favorable prognosis should be treated more aggressively [7]. Although allogeneic hematopoietic stem cell transplantation (HSCT) for patients with ALL in CR1 is much more intensive than multi-agent combined chemotherapy, it is associated with increased morbidity and mortality when compared with such chemotherapy.

Minimal residual disease (MRD) status has been proven to be a strong prognostic factor for adult patients with Ph-negative ALL [8-14]. In this study, we prospectively monitored the MRD status after CR induction and consolidation chemotherapies in adult patients with Ph-negative ALL to determine the clinical indications for allogeneic HSCT.

## Patients & methods

### Patient eligibility criteria

A total of 110 adult ALL patients were enrolled in this study between July 2002 and August 2008 on the basis of the following eligibility criteria: non-L3 ALL, 16–65 years of age, an Eastern Cooperative Oncology Group performance status of 0–2, and adequate liver and kidney function (serum bilirubin, ≤2.0 mg/dl and serum creatinine, ≤2.0 mg/dl, respectively). Cytogenetic studies were performed on pretreated bone marrow or unstimulated blood samples using the standard banding technique. The treatment protocol was approved by the institutional review board of each participating hospital. Written informed consent was obtained from all patients in accordance with the Declaration of Helsinki. Of the 110 patients enrolled, 42 were excluded from the study because of Ph-positivity, 5 because of misdiagnosis, 2 because of infectious complications, and 1 each because of liver damage and protocol violation. The remaining 59 patients were Ph*-*negative.

### Treatment

We used a modified CALGB 19802 [15,16] treatment protocol that comprised 6 courses of chemotherapy administered in the order of A-B-C-A-B-C regimens, followed by a maintenance phase. Induction chemotherapy (course A) was a 21-day course consisting of cyclophosphamide (CPM; 1200 mg/m^2^ on day 1), daunorubicin (DNR; 60 mg/m^2^ on days 1, 2, and 3), vincristine (VCR;1.3 mg/m^2^ [maximum 2 mg] on days 1, 8, 15, and 22), L-asparaginase (3000 U/m^2^ on days 9, 11, 13, 16, 18, and 20), and prednisolone (PSL; 60 mg/m^2^ [max 100 mg]). Granulocyte-colony stimulating factor (nartograstim) was administered starting from day 4 and continued until neutrophil recovery. For patients aged 55 years or older, the doses of CPM and DNR were reduced to 500 mg/m^2^ and 50 mg/m^2^, respectively. Furthermore, PSL therapy was shortened to 7 days in these patients. The first consolidation therapy (course B) consisted of mitoxantrone (MIT; 10 mg/m^2^ on days 2 and 3), cytarabine (AraC; 2000 mg/m^2^/day on days 1, 2, 3, and 4) and intrathecal administration of methotrexate (MTX; 15 mg/body on day 1). For patients aged 55 years or older, the doses of MIT and AraC were reduced to 7 mg/m^2^ and 1500 mg/m^2^/day, respectively. The second consolidation therapy (course C) consisted of VCR (1.3 mg/m^2^ [max 2 mg] on days 1, 8, and 15) and MTX (1500 mg/m^2^ on days 1, 8, and 15) with leucovorin rescue and intrathecal MTX on days 1, 8, and 15. The patients received the following maintenance chemotherapy on a monthly basis: PSL, 60 mg/m^2^ on days 1–5; VCR, 1.3 mg/m^2^ (max 2 mg) on day 1; oral MTX, 20 mg/m^2^ weekly; and oral 6-mercaptopurine, 60 mg/m^2^ daily. MRD status was evaluated after the induction therapy (first course A) and after the second consolidation therapy (first course C). Patients with positive MRD following the second consolidation therapy were considered to be indicated for allogeneic HSCT as soon as possible. Eligible donors included HLA-identical related, HLA-identical unrelated donors from Japan Marrow Donation Program, and cord blood from Japan Cord Blood Bank Network. Conditioning before allogeneic HSCT and prophylaxis for graft-versus-host disease was performed according to each institutional standard.

### MRD analysis

#### Real-time quantitative polymerase chain reaction (RQ-PCR) analysis of chimeric mRNA

mRNA from bone marrow cells were analyzed for the presence of major and minor *BCR/ABL, TEL/AML1, MLL/AF4, MLL/AF9, MLL/AF6, MLL/ENL, E2A/PBX1*, and *SIL/TAL1* chimeric genes. Samples were amplified by RQ-PCR and quantified by parallel amplification of serial dilutions of transcript-containing plasmids [17,18].

#### PCR analysis of *TCR/Ig* rearrangement

High-molecular weight DNA from marrow cells was initially screened for major rearrangement patterns of *TCRγ, TCRδ,* and *Igκ*, and secondarily screened for rearrangements in *Ig heavy chain* (*IgH*), using previously described primers [19-21]. Two-step (nested) PCR for MRD quantification was performed using allele-specific oligonucleotide (ASO)-primers based on the sequence of PCR screening products, which had clonal recombinations by heteroduplex analyses. Prior to PCR analysis, DNA samples from post-treatment bone marrow samples and DNA from the samples obtained at diagnosis were serially diluted (between 10^−2^ and 10^−5^) with buffy coat DNA from eight healthy volunteers. Buffy coat DNA was also used as a control for nonspecific amplification of comparable *Ig/TCR* arrangements present in normal cells. All PCR reactions were performed simultaneously and analyzed using ethidium staining and agarose gel electrophoresis. MRD was quantified by comparing the intensities of band signals on an agarose gel stained with ethidium bromide without amplification of the background. MRD quantifications were performed using ASO-primers with a sensitivity of ≤1 × 10^−4^, and MRD positivity was defined as a lower limit of detection of ≥1 × 10^−3^.

#### Statistical analysis

Statistical analyses of the data accumulated throughout October 2011 were performed. Overall survival (OS) was defined as the time between diagnosis and the end of the trial or death, and disease-free survival (DFS) was defined as the time from CR to relapse or death while still in CR. Survival curves were estimated using the Kaplan–Meier method, and the statistical significance of differences in survival was determined using the log-rank test.

The influence of prognostic factors including age, white blood cell (WBC) count, and MRD status on DFS was estimated with multivariate Cox regression analysis. The level of statistical significance was set at 0.05.

## Results

### Treatment outcome

The median follow-up time was 1134 days (range, 14–3248 days). A total of 59 patients were Ph-negative (29 males and 30 females), and their median age was 35 years ranging from 16 to 63. The median white blood cell count at presentation was 11.0 × 10^3^/L (range 0.9–409). CR was achieved in 47 patients (80%). Six patients died during induction; their causes of death included sepsis (n = 3), pneumonia (n = 2), and other (n = 1). There were 29 survivors after the median follow-up period. The probability of 3-year OS and DFS in these patients with Ph-negative ALL was 59% and 52%, respectively (Table 1).

**Table 1:** Patient characteristics and clinical outcome

	**Ph negative**	**Ph negative & known MRD status**
Total No. patients	59	43
Sex, No. (%)		
Male	29 (49)	21 (49)
Female	30 (51)	22 (51)
Median Age, (range)	35 (16-63)	31 (17-63)
Median WBC count, ×10^9^/L, (range)	11.0 (0.9-409)	10.6 (1-409)
Immunophenotype, No. (%)		
B-lineage	45 (76)	36 (84)
T-lineage	14 (24)	7 (16)
CR rate, No. (%)	47 (80)	39 (91)
3-years OS (%)	59	74
3-years DFS (%)	52	56

*MRD*, minimal residual disease; *Ph*, Philadelphia chromosome; *CR*, complete remission; *OS*, overall survival; *DFS*, disease-free survival.

### Relationship between MRD status and treatment outcomes

**Figure 1:**
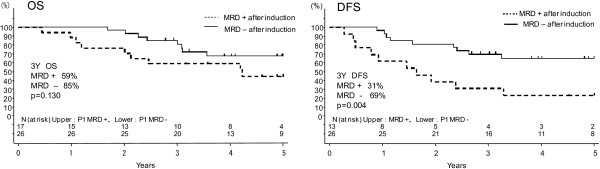
**Impact of post-induction minimal residual disease (MRD) status on overall survival (OS) and disease-free survival (DFS). **Patients who were MRD negative after induction therapy (first course A) (n = 26) had a significantly better 3-year DFS compared with those who were MRD positive (n = 13) (69% vs. 31%, p = 0.004).

Among the 59 Ph-negative ALL patients, 43 patients (73%) could be monitored for MRD status, and the remaining 16 patients were not because 10 had no clonal *TCR/Ig* targets or chimeric mRNA and 6 did not provide sufficient DNA or RNA from their samples. The MRD status of 43 patients (21 males and 22 females; median age: 31 years, ranging from 17 to 63; median WBC count at presentation: 10.6 × 10^3^/L ranging 1–409) was determined by PCR analysis of major gene rearrangements and/or chimeric mRNAs (15 were positive for *TCRγ*, 6 for *TCRδ*, 6 for *Igκ*, 11 for *IgH*, 1 for *TCRγ* and *TCRδ*, 1 for *TCRδ* and *IgH*, 1 for *E2A-PBX*, 1 for *MLL-AF4*, and 1 for *MLL-ENL*). CR was achieved in 39 of these 43 patients with known MRD status (91%). The median follow-up time was 1421 days (range, 162–3248 days). The probability of 3-year OS and DFS in the Ph-negative patients with known MRD status was 74% and 56%, respectively (Table 1). In terms of CR1 status, MRD-negative patients after induction chemotherapy A in the first course (n = 26) showed a better 3-year DFS (69%) compared with MRD-positive patients (n = 13; 31%), as shown in Figure 1. The difference was statistically significant (p = 0.004). MRD-negative patients also showed a significantly lower 3-year relapse rate compared with MRD-positive patients (28% vs. 58%, p = 0.031).

**Figure 2:**
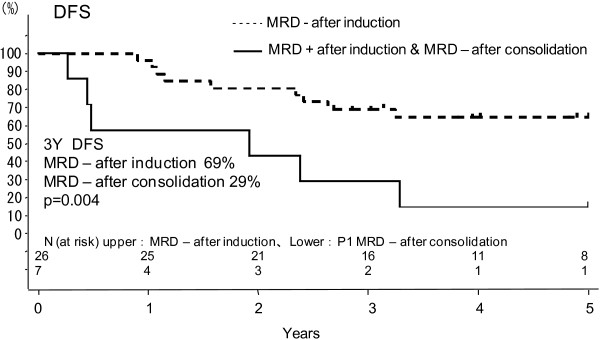
**Effect of time taken to become minimal residual disease (MRD)-negative on disease-free survival (DFS). **Patients who were MRD positive after induction and became MRD negative after consolidation chemotherapy C in the first course (n = 7) had a significantly worse DFS compared with patients who were MRD negative after induction chemotherapy A in the first course (n = 26) (69% vs. 29%, p = 0.004).

There was no patient who proceeded to allogeneic HSCT among 26 MRD-negative patients after induction therapy in CR. In contrast, patients who were MRD-positive after induction but became MRD-negative after consolidation chemotherapy C in the first course (n = 7) showed a significantly worse 3-year DFS compared with patients who were MRD-negative after induction chemotherapy A in the first course (29% vs. 69%, p = 0.004), as shown in Figure 2. Among 7 late-attained MRD-negative patients, three patients proceeded to allogeneic HSCT when MRD status became positive again under maintenance therapy. Six patients were MRD-positive after consolidation chemotherapy C in the first course, and 3 patients among them proceeded to allogeneic HSCT, while other 3 patients did not because of lack of a suitable donor (n = 1) and of patients’ refusal to allogeneic HSCT (n = 2). All of 3 MRD-positive patients who did not undergo allogeneic HSCT, relapsed and died within 3 years after CR, whereas 2 of 3 patients those who received allogeneic HSCT gave DFS at 3 years. Table 2 shows the results of multivariate Cox regression analysis for DFS in 43 MRD-evaluable patients. The analysis indicates that age (≥35 years vs. <35 years: Hazard ratio (HR) 5.067, and p = 0.005) and MRD status after induction therapy (positive vs. negative: HR 8.769, and p < 0.001) were significant prognostic factors, whereas WBC count (≥30 × 10^9^/L vs. <30 × 10^9^/L: HR 1.496, and p = 0.505) or MRD status after consolidation therapy (positive vs. negative: HR 0.675, and p = 0.556) was not.

**Table 2:** Multivariate analysis for disease-free survival (Cox Regression Model)

	**Hazard ratio**	**95% CI**		**P-value**
Risk factors				
Age	5.067	1.616	15.885	0.005
WBC	1.496	0.457	4.897	0.505
MRD status after induction	8.769	2.465	31.196	<0.001
MRD status after consolidation	0.67	0.18	2.492	0.55

Age: ≥35 vs <35.

WBC: ≥30×10^9^/L vs <30×10^9^/L.

MRD after Induction: positive vs. negative.

MRD after consolidation: positive vs. negative

## Discussion

Compared with treatments for childhood ALL, those for adult ALL are far less effective [22], and allogeneic HSCT is frequently recommended as the most potent post-remission therapy for ALL patients in CR1 [23]. Since relapse in ALL patients leads to very poor prognosis [24-26], the notion that allogeneic HSCT should be performed for all patients with ALL beyond CR1 is difficult to be realized in clinical situations [7].

The international ALL trial MRC UKALL XII/ECOG E2993 showed that allogeneic HSCT using matched related donors provided survival benefit for standard-risk adult patients with Ph-negative ALL in CR1 compared with chemotherapy, while there was no significant survival benefit for high-risk patients. Allogeneic HSCT is able to reduce relapse rates in both standard-risk and high-risk patients; however, there is a decrease in OS in the high-risk patients because of their higher rates of transplant-related mortality. The high-risk in this international study was defined as having as 1 of the following factors: age more than 35 years, a high WBC count at presentation (>30 × 10^9^/L for B lineage and >100 × 10^9^/L for T lineage) [23]. Age is a significant prognostic factor for ALL patients receiving allogeneic HSCT as well as chemotherapy [27]. Therefore, allogeneic HSCT may not be a recommended option for patients defined as high-risk because of their age being more than 35 years old [28,29].

Recent studies have shown that a pediatric-inspired ALL chemotherapy protocol significantly improves treatment outcome in relatively young adult ALL patients [30-34], and this patient population is at standard-risk in terms of age. Thus, the indication of allogeneic HSCT based on the risk stratification made by initial presentation needs to be tested, and more reliable indication for allogeneic HSCT in adult patients with Ph-negative ALL in CR1 is necessary.

MRD measurement in adult patients with Ph-negative ALL has been reported to be useful for identifying patients with a significantly high risk of relapse. The German Multicenter Study Group for adult ALL (GMALL) study used PCR analysis of antigen-receptor genes to assess MRD in standard-risk ALL patients. Low-risk patients were those with MRD–negative on days 11 and 24 and had a 3-year relapse rate of 0%; high-risk patients were those with MRD-positive until week 16 and had a relapse rate of 94% [10]. The Northern Italy Leukemia Group-ALL 09/00 study found that MRD was the most significant predictor of relapse [13]. The estimated 5-year DFS was 72% in 58 MRD-negative patients at the end of consolidation and 14% in 54 MRD-positive patients.

Our results indicate that patients with MRD negativity after induction therapy provided excellent DFS without allogeneic HSCT, whereas patients with MRD positivity after several consolidation therapies showed very poor DFS if they did not undergo allogeneic HSCT. This observation is in line with above reports. Our analysis showed that late-attained MRD negativity could not lead to good prognosis, while other groups reported the MRD negativity at the end of consolidation to be associated with good prognosis. This controversy may reflect the sensitivity level of MRD measurement. We used semi-quantitative PCR analysis and defined MRD negativity as <1 × 10^−3^, whereas GMALL [10] and The Northern Italy Leukemia Group-ALL 09/00 study [13] analyzed MRD according to EuroMRD-ALL guidelines [35,36] and considered <1 × 10^−4^ as MRD negativity. In our study, MRD positivity after the second course of consolidation was seen in 6 of 37, while MRD positivity at the end of consolidation was observed in 54 of 112 in The Northern Italy Leukemia Group-ALL study 09/00 [13]. Thus, our MRD-negative patients had a possibility of MRD positivity if more sensitive MRD analysis was used. We suggest these late-attained MRD-negative patients were potential candidates for allogeneic HSCT.

In this study, the results of multivariate Cox regression analysis for DFS indicates that age (≥35 years vs. <35 years) and MRD status after induction therapy were significant prognostic factors, whereas WBC count (≥30 × 10^9^/L vs. <30 × 10^9^/L) or MRD status after consolidation therapy was not. Age is one of the most important prognostic factors in adult ALL patients, and age of our study population was median 31 years-old ranging 17 to 63 including adolescent and young adult patients. Thus, these relative young patients were supposed to have good prognosis with chemotherapy. Initial WBC count has been another important prognostic factor in adult ALL patients, but not in our study. Our chemotherapy regimen was modified CALGB 19802 with dose intensification of daunorubicin and cytarabine, and it might be possible that this intensive chemotherapy conquer negative impact of high initial WBC count. According to the recently reported result of CALGB 19802 [16], age (≥60 years vs. <60 years) was a significant prognostic factor for DFS, while initial WBC count (≥30 × 10^9^/L vs. <30 × 10^9^/L) was not. This report was in line with our observation. In our analysis MRD status after induction therapy (positive vs. negative: HR 8.769, and p < 0.001) was a very strong prognostic factor for DFS. Whether negative impact of MRD positivity could be overcome by allogeneic HSCT is the next consideration. There are two reports regarding the effect of prospective allocation for allogeneic HSCT based on MRD positivity in adult patients with Ph-negative ALL in CR1.

In the Northern Italy Leukemia Group-ALL study 09/00, for the MRD-positive patients at the end of consolidation, there was a significantly better 4-year DFS for 36 patients who had an allogeneic (n = 22) and autologous (n = 14) HSCT compared to 18 patients unable to undergo HSCT (33% vs. 0%, p = 0.0000) [13]. The GMALL reported that 5-year DFS for MRD-positive patients at week 16 with (n = 57) vs. without (n = 63) allogeneic HSCT were 44 ± 8% vs. 11 ± 4% respectively (p <0.0001) [37]. In our study, among MRD-positive patients following consolidation chemotherapy C in the first course, all of 3 patients without allogeneic HSCT relapsed while 1 of 3 patients with allogeneic HSCT did. The size of our study population was too small for statistical analysis. However, these three studies clearly indicate that MRD-positive patients at late phase of chemotherapy have little chance of DFS more than 10% without allogeneic HSCT [37]. These MRD-defined high-risk patients had much worse prognosis compared with conventional high-risk patients defined by initial presentation. Furthermore, allocation of allogeneic HSCT could improve the prognosis of MRD-defined high-risk patients.

The interpretation of our results may be affected by a limited number of adult ALL patients. A role of MRD measurement should be evaluated in relation with patients’ geography, chemotherapy regimens used, and timing and sensitivity of MRD analysis. With our less sensitive MRD analysis compared to EuroMRD-ALL guidelines, we could identify patients with good early treatment response not indicated for allogeneic HSCT, while we could not identify patients with good late treatment response. In near future, the assessment of MRD status using standardized protocols and RQ-PCR [35,36] will be a valuable tool to stratify a risk of relapse in adult patients with Ph-negative ALL in CR1.

In conclusion, our data suggest that evaluation of MRD at least twice after induction and consolidation is very useful when considering clinical indication for allogeneic HSCT in adult patients with Ph-negative ALL in CR1.

## Competing interests

The authors declare no competing financial interests.

## Authors’ contributions

All authors recruited and treated patients for this study. DNA-baed MRD analysis in this report were performed under supervision of SY. KN and MH were involved in the drafting of the manuscript. MH coordinated the study. All authors reviewed and approved the final draft of the manuscript.
